# Postpartale Keratopathie

**DOI:** 10.1007/s00347-020-01315-y

**Published:** 2021-01-08

**Authors:** J. Jakob-Girbig, D. Meller

**Affiliations:** Universitätsklinikum Jena, Am Klinikum 1, 07747 Jena, Deutschland

## Falldarstellung

Keratopathien mit untypischem klinischem Verlauf und wiederholten Wundkontaminationen sollten an das Vorliegen von Selbstverletzung denken lassen. Vor allem in der sehr vulnerablen postpartalen Phase ist es notwendig, im Falle einer entsprechenden Befundkonstellation umgehend einen interdisziplinären Therapieansatz mit integrierter psychiatrischer Mitbetreuung zu ermöglichen.

### Anamnese

Eine 25-jährige Patientin entwickelte ca. 2 Wochen nach komplizierter Spontangeburt mit begleitendem HELLP-Syndrom eine streng einseitige Durchwanderungskeratitis mit zentralem Hornhautulkus des linken Auges. Sie wurde daraufhin extern bei Nachweis koagulasenegativer Staphylokokken im Bindehautabstrich antibiogrammgerecht mittels Chloramphenicol-Augensalbe und Levofloxacin-Augentropfen rezidivierend stationär behandelt. Bei zunehmender Befundprogredienz mit Visusabfall auf Handbewegungen wurde uns die Patientin 2 Monate postpartal zuverlegt.

### Klinischer Befund

**Figure 1:**
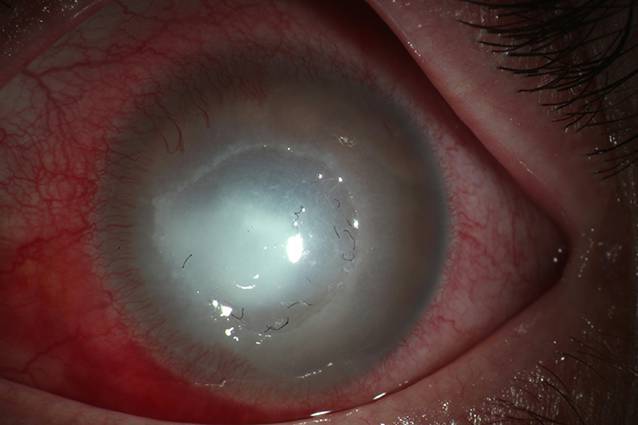

Es konnte am rechten Auge ein Visus von 0,2 LogMAR und am linken Auge von Lichtscheinprojektion ermittelt werden. Der Vorderabschnitt rechts zeigte mit einer glatten, klaren Hornhaut, reizfreier Bindehaut, fehlendem Vorderkammerreiz und altersentsprechender Linse einen unauffälligen Befund. Auch der Fundus rechts entsprach einem altersentsprechenden Normalbefund. Der Vorderabschnitt des linken Auges hingegen zeigte eine extreme Injektion der Bindehaut, eine in toto getrübte Hornhaut mit einem flächigen zentralen Ulkus und einer mitteltiefen Vorderkammer ohne sichtbare Details. Auffällig waren multiple schwarze Anlagerungen eines stoffähnlichen Materials im Bereich der Hornhaut (Abb. 1). Sonographisch konnten eine zirkulär anliegende Netzhaut und ein infiltratfreier Glaskörper dargestellt werden.

### Verlauf

Zur stationären Aufnahme wurde das stoffähnliche Material von der Hornhaut links entfernt und ein erneuter Bindehautabstrich genommen. Es wurde Gentamycin subkonjunktival appliziert, die übrige Lokaltherapie vorerst belassen und eine Augenklappe verordnet. Die Patientin wurde darauf hingewiesen, am Auge nicht zu reiben und die Augenklappe zu belassen.

**Figure 2:**
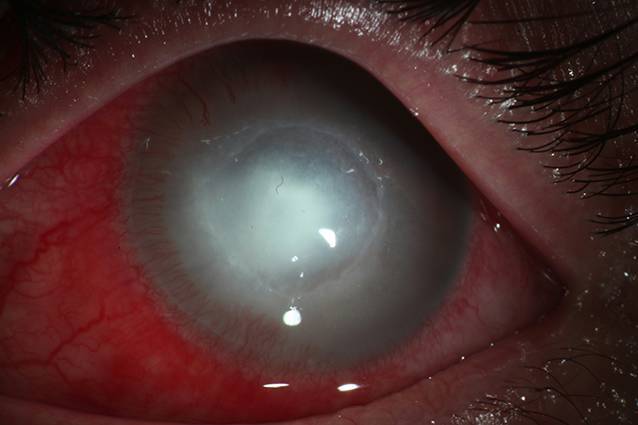

Am nächsten Tag zeigte sich ein stabiler Befund ohne weiteres Flusenmaterial (Abb. 2).

**Figure 3:**
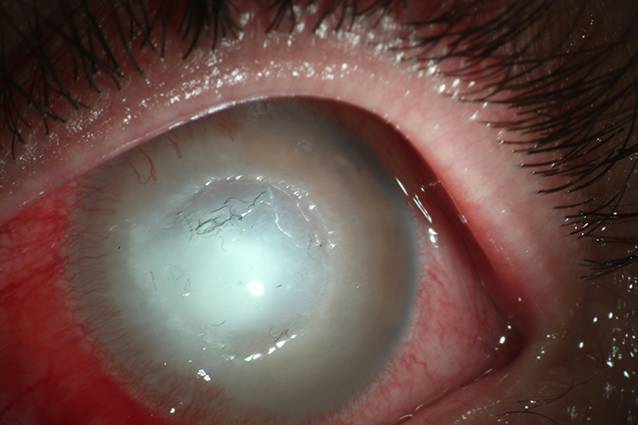

Am Folgetag jedoch fanden sich erneut massive Anlagerungen am linken Auge und eine beginnende Einschmelzung des Ulkus (Abb. 3). Im Kontakt wirkte die Patientin seit Aufnahme affektverflacht und wenig zugewandt. Fragen wurden von ihr nur sehr knapp beantwortet, und Blickkontakt war kaum möglich. In Zusammenschau der Befunde wurde der Verdacht auf eine Manipulation ihrerseits immer wahrscheinlicher, sodass der psychiatrische Konsildienst hinzugezogen wurde. Hierbei wurde der Verdacht auf eine depressive Episode bzw. auf eine artifizielle Störung geäußert. Die Patientin lehnte allerdings entsprechende therapeutische Maßnahmen ausdrücklich ab. Nach Eingang des mikrobiologischen Befundes erfolgte eine Umstellung der Lokaltherapie auf Gentamycin- und Ofloxacin-Augentropfen, und es wurden Gentamycin und zusätzlich Dexamethason subkonjunktival appliziert.

Im weiteren Verlauf kam es zum Fortschreiten des Ulkusbefundes mit rezidivierenden flusenartigen Anlagerungen. Unter Einbeziehung des Ehemannes und intensiver Gesprächsangebote psychiatrischerseits räumte die Patientin eine zunehmend gedrückte Stimmung seit der Geburt ihres Kindes ein, wobei die Manipulation am Auge weiterhin verneint wurde. Somit wurde die Diagnose einer postpartalen Depression mit begleitendem selbstverletzendem Verhalten gestellt. Die Lokaltherapie war bei fortschreitendem Befund zwischenzeitlich um Voriconazol Augentropfen erweitert worden.

Schließlich stimmte die Patientin einer antidepressiven medikamentösen Behandlung zu, und eine orale Therapie mit Sertralin wurde eingeleitet. Hierunter kam es zu einer raschen Stabilisierung der allgemeinen Stimmungslage der Patientin, und auch die flusenartigen Anlagerungen am linken Auge waren nicht mehr nachweisbar.

Bei therapieresistenter Erosio corneae links wurde im Verlauf 2‑malig eine Amnionmembrandeckung erforderlich, die komplikationslos durchgeführt wurde. Nach 40 Tagen stationärem Aufenthalt wurde die Patientin in die Häuslichkeit entlassen.

**Figure 4:**
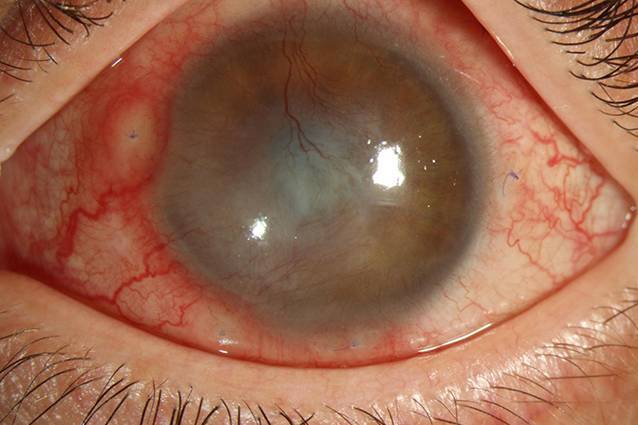

Zur bisher letzten ambulanten Befundkontrolle zeigte sich am linken Auge eine in toto vaskularisierte Hornhaut mit ausgeprägter zentraler Eintrübung und verschlossenem Epithel (Abb. 4). Der Visus lag bei Handbewegungen. Es wurde eine Lokaltherapie mit Dexa Sine Augentropfen und hyaluronsäurehaltigen Pflegepräparaten ordiniert.

Im Verlauf soll über die Durchführung keratoregenerativer Eingriffe wie Limbusstammzelltransplantation und Keratoplastik entschieden werden.

## Diskussion

Augenverletzungen stellen eine ungewöhnliche, aber sehr wichtige Form von selbstverletzendem Verhalten dar [1]. Dabei findet sich ein breites Spektrum möglicher Verletzungsarten, das von milden Vorderabschnittstraumata bis hin zur Selbstenukleation reicht [2]. Am häufigsten werden die eigenen Finger zur Beibringung der Verletzungen genutzt, aber auch Instrumente wie Scheren, Messer [3] oder Rasierklingen [4] kommen zum Einsatz. Mögliche Ursachen dieses Verhaltens sind Schizophrenien, durch Substanzmissbrauch induzierte Psychosen, Zwangsstörungen, mentale Retardierung, rituelle Praktiken, organische Erkrankungen, wie z. B. Neurosyphilis oder strukturelle Hirnverletzungen, Depressionen [2] und auch Autismus [5].

Die ersten 12 Monate nach der Geburt eines Kindes stellen für jede Mutter eine sehr vulnerable Phase dar, innerhalb derer es zu ausgeprägten körperlichen, seelischen und sozialen Veränderungen kommt [6]. Vor allem innerhalb der ersten 3 Monate postpartal ist die Inzidenz neu auftretender psychischer Erkrankungen hoch [7]. So leiden 8–15 % der Neumütter unter postpartalen Depressionen [8]. Frauen, die postpartal Symptome von Depression oder Angststörung zeigen, haben ein erhöhtes Risiko für selbstverletzendes Verhalten [9]. Sollte dies auftreten, erhöht sich wiederum das Risiko eines späteren Suizides [10].

Im vorliegenden Fall führte eine postpartale Depression zu einem schweren selbstverletzenden Verhalten, welches sich gegen das linke Auge der Patientin richtete. Erst durch den untypischen Krankheitsverlauf und die sich wiederholende Kontamination des Auges mit Stoffpartikeln wurde der Verdacht auf Selbstverletzung gestellt. Da sich auch das übrige Verhalten der Patientin im sozialen Kontext als auffällig erwies, konnte unter psychiatrischer Mitbeurteilung schließlich die Diagnose einer postpartalen Depression mit begleitendem selbstverletzendem Verhalten gestellt und die Patientin einer entsprechenden Therapie zugeführt werden.

In der Literatur konnten keine weiteren Beispiele für selbst beigebrachte Augenverletzungen im Rahmen einer postpartalen Depression gefunden werden. Aufgrund der Schwere des Krankheitsbildes und der möglichen Folgekomplikationen, v. a. im Hinblick auf die erhöhte Suizidrate, ist es aus unserer Sicht sehr entscheidend, diese mögliche Diagnose zu kennen und bei entsprechenden Befundkonstellationen in differenzialdiagnostische Überlegungen einzubeziehen.

## Fazit für die Praxis

- Erkrankungen von Müttern innerhalb der postpartalen Phase bedürfen einer besonderen Aufmerksamkeit.
- Untypische Krankheitsverläufe und sich wiederholende Wundkontaminationen sollten an selbstverletzendes Verhalten denken lassen.
- Bestätigt sich der Verdacht auf selbst beigebrachte Augenverletzungen, ist es empfehlenswert, sich an die entsprechenden psychiatrischen Kollegen zu wenden.
- Die Therapieplanung und -durchführung sollte interdisziplinär erfolgen.
